# Oncogenic role of the chromobox protein CBX7 in gastric cancer

**DOI:** 10.1186/1756-9966-29-114

**Published:** 2010-08-19

**Authors:** Xiao-Wei Zhang, Li Zhang, Wei Qin, Xiao-Hong Yao, Lei-Zhen Zheng, Xin Liu, Jin Li, Wei-Jian Guo

**Affiliations:** 1 Department of Medical Oncology, Fudan University Shanghai Cancer Center; Department of Oncology, Shanghai Medical College, Fudan University, shanghai 200032, China; 2 Department of Medical Oncology, Xinhua Hospital, School of Medicine, Shanghai Jiaotong University, Shanghai 200092, China

## Abstract

**Background:**

Chromobox 7 (CBX7) is a Polycomb family protein that extends the lifespan of normal human cells via downregulating the expression of *INK4a/ARF *tumor suppressor locus. It was found that CBX7 expression was upregulated in lymphoma, but downregulated in some other human malignancies. The role of CBX7 in most types of cancer is still not clear. The purpose of this study is to investigate the role of CBX7 in gastric cancer.

**Methods:**

The expression of CBX7 and its potential target protein p16(INK4a) in gastric cancer cell lines and gastric tumors was assayed by Western blot analysis and immunohistochemistry(IHC). The correlations between CBX7 expression and p16(INK4a), clinicopathological characteristics, and prognosis were analyzed. Gastric cancer cell line SGC-7901 was transfected with CBX7 siRNA expressing plasmids, and the expression of various proteins was analyzed by Western blot analysis. Cellular senescence, anchorage independent growth, and cell migration assays were performed to determine the functional role of CBX7 in gastric cancer cells.

**Results:**

CBX7 was found to be overexpressed in gastric cancer cell lines and gastric tumors. Overexpression of CBX7 in gastric cancer tissues correlated with patients' age, clinical stage and lymph node metastasis. Knockdown of CBX7 expression in gastric cancer cells led to increased cellular senescence, decreased cellular proliferation and migration ability, accompanied by upregulation of p16(INK4a).

**Conclusions:**

CBX7 acts as an oncogene in the carcinogenesis and progression of gastric cancer, and it may regulate tumorigenesis, cell migration and cancer metastasis partially via p16(INK4a) regulatory pathway.

## Background

Polycomb group (PcG) proteins are a class of epigenetic regulators, which always form multiprotein complexes to exert their functions in regulating cell proliferation, senescence and tumorigenesis via well-known growth regulatory pathways [1]. More and more studies have implicated the deregulation of different PcG proteins in carcinogenesis and neoplastic progression. Bmi-1 is one of the best known PcG gene, which was initially identified for its ability to cooperate with c-Myc in lymphomagenesis and subsequently was found to be overexpressed in many kinds of human cancers and thus was accepted as an oncogene [2-10]. Overexpression of Bmi-1 has been shown to immortalize and transform normal human cells via inhibiting cellular senescence, which constitutes a powerful barrier to oncogenesis [8,11]. *INK4A/ARF *tumor suppressor locus is one of the most important cancer relevant targets of Bmi-1. We have found that regulation of AKT/PKB pathway is another important mechanism for Bmi-1 in breast and gastric cancers [8,10].

CBX7, another PcG protein, shares no homology with Bmi-1 but was found to have similar functions and mechanisms as Bmi-1 that inhibits cellular senescence and extends the lifespan of normal human cells via downregulating the expression of *INK4a/ARF *locus, and cooperates with c-Myc in lymphomagenesis [7,8,11]. These data suggested that CBX7 functions as an oncogene like Bmi-1. However, several recent studies showed that decrease or loss of CBX7 protein expression correlated with a more aggressive phenotype in pancreatic, thyroid and colorectal cancer, which suggested that CBX7 might act as a potential tumor suppressor [12-14]. The results are controversial and the functions and mechanisms of CBX7 in caicinogenesis are still far from clear. The opposite expression level of CBX7 in different studies may due to the different cancer types. Its role in different cancer types and different pathological conditions needs to be clarified. Regulation of *INK4a/ARF *locus by CBX7 also needs further confirmation in cancer cells.

Gastric cancer is one of the most common malignancies throughout the world, and mechanisms that underlie the carcinogenesis of gastric cancer are still poorly understood. Recently we found that Bmi-1 plays an important role in the carcinogenesis and progression of gastric cancer and acts as an oncogene [10]. Does CBX7 also play a role in the carcinogenesis and progression of gastric cancer needs to be studied. One newly published paper revealed that CBX7 might be negatively regulated by miRNA421 in gastric cancer cell line [15], though the expression and function of CBX7 in gastric cancer are still unclear. Here, we show that CBX7 is overexpressed in gastric cancer cell lines and gastric cancer tissues, and its expression correlates with patients' age, clinical stage, lymph node metastasis, and poor prognosis. We also report that knockdown of CBX7 expression in gastric cancer cell lines results in induction of a senescence-like phenotype and reduction of transformed properties, which is accompanied by upregulation of p16(INK4a). These data suggest that CBX7 may act as an oncogene in gastric cancer partially via regulation of p16(INK4a).

## Methods

### Cellular reagents, molecular reagents, and methods

One immortalized human gastric mucosal epithelial cell line (GES-1) and eight human gastric cancer cell lines (MKN28, MKN45, KATOIII, NCI-N87, SNU-1, SNU-16, SGC-7901, AGS) were preserved in Surgical Institution of Ruijin Hospital. These cell lines were cultured in RPMI-1640 supplemented with 10% fetal bovine serum (FBS) and antibiotics. CBX7 short interfering RNA (siRNA) was designed and cloned in the retroviral vector pGCL-GFP obtained from GeneChem Inc. (Shanghai, China). The sequence of CBX7 siRNA (CBX7 i) was as follows: CACCTTGCATGCACCTTGCTA. Nonsilencing (NS)-siRNA was used as a control(Ctrl i). The retroviruses were produced by transient transfection of the retroviral vector together with pIK packaging plasmid into 293 packaging cell line as described, and stable cell lines expressing CBX7 i (CBX7 siRNA) or Ctrl i (control siRNA) were generated by infection of the retroviruses as described [16]. The senescence in gastric cancer cells was determined by senescence-associated beta galactosidase (SA-β-gal) assay as described [17]. Soft-agar assay to determine the anchorage independent growth of cells was done as described [18]. Transwell chamber (Corning Costar, Cambridge, MA) migration assay was performed as described [18] to detect cell migration ability.

### Clinical samples

Seventy five paraffin-embedded human gastric cancer tissue samples were collected from the archives of the department of pathology for further immunohistochemical analysis of different proteins' expression. These patients were diagnosed as gastric cancer and received treatment in Xinhua hospital during 1999 and 2000. Sixty nine patients received radical surgery, and followed by 5-Fu based postoperative ajuvant chemotherapy for patients with advanced stage(T3/4 or N1-3). Six patients were found to have liver or peritoneal metastases during operation and received palliative operation, followed by 5-Fu based palliative chemotherapy. The clinicalpathologic variables were obtained from the medical records and the disease stages of the patients were classified according to the 2002 UICC gastric cancer TNM staging system. For the use of these clinical materials for research purposes, prior patients' consent and approval from the Institute Research Ethics Committee was obtained.

### Immunological reagents, Western blot, and Immunohistochemical analyses

CBX7 was detected by using a rabbit polyclonal antibody from Abcam (Cambridge, UK), and p16(INK4a) was detected by a mouse monoclonal JC8 (Santa Cruz Biotech, CA). Western blot analyses to detect the expression of CBX7, p16(INK4a), and β-actin proteins in gastric cancer cell lines were performed as described[16]. Immunohistochemical (IHC) analyses to detect the expression of CBX7, and p16(INK4a) in paraffin sections were performed as described [19]. All slides were interpreted by two independent observers in a blinded fashion. More than 10% of the cells were stained with moderate or strong staining intensity was considered positive. Otherwise, the sample was considered negative.

### Statistical analysis

All statistical analyses were done by using the SPSS 15.0 software package. In the set of IHC assay of paraffin-embedded tissue samples, the Pearson χ^2 ^test was used to estimate the correlations between CBX7 and p16(INK4a), and clinicopathologic characteristics. Cumulative survival curves were plotted by the Kaplan-Meier method and the relationship between each of the variables and survival was assessed by Log-rank test in univariate analysis. The parameters were then tested by multivariate Cox proportional hazards model, which was performed to identify independent variables for predicting survival. A p value less than 0.05 was considered statistically significant. In *In vitro *experiments, data was described as mean ± SD, and analyzed by Student's t-test.

## Results

### Overexpression of CBX7 in gastric cancer cell lines and gastric tumor tissues

**Figure 1:**
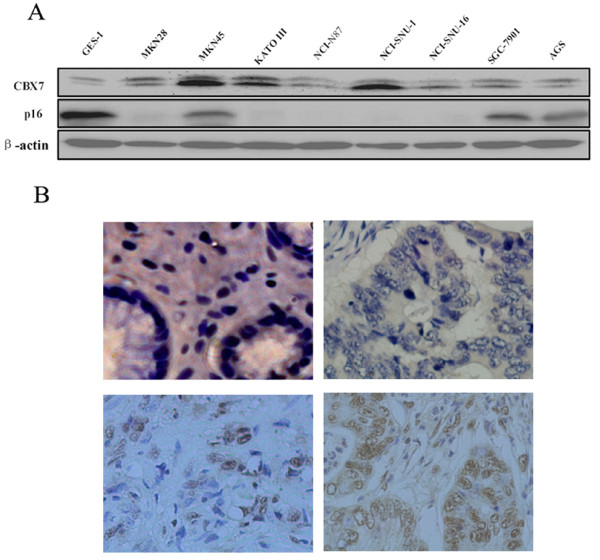
**The expression of CBX7 in gastric cancer cell lines and gastric tumors. A) **The expression of CBX7 and p16 proteins in an immortalized human normal gastric epithelial cell line GES-1 and various gastric cancer cell lines as detected by Western blot analysis. β-actin was used as a loading control. **B) **Examples of nuclear staining of CBX7 in normal gastric tissues and gastric cancer tissues by IHC detection: negative CBX7 expression in normal gastric tissue (upper left); negative CBX7 expression (upper right), slight positive CBX7 expression (lower left), and strong CBX7 expression (lower right) in gastric cancer tissues. Tissue sections were stained with CBX7-specific antibody and counterstained with hematoxylin as described in experimental procedures (magnification: ×400).

Firstly, we analyzed the expression of CBX7 in several gastric cancer cell lines by western blot. Our results showed that compared to GES-1, a normal immortal human gastric mucosal epithelial cell line, 3 out of 8 gastric cancer cell lines expressed obviously high CBX7 at protein level (Fig 1A). Then, we studied the expression of CBX7 in normal gastric tissues and gastric tumor tissues by IHC (Fig 1B). By IHC analysis, 25 of 75 (33.3%) paraffin-embedded archival gastric tumor biopsies showed a positive staining for CBX7. These sections examined contained adjacent normal gastric tissue in 60 cases, and only 1 of them (1/60, 1.7%) showed positive staining of CBX7. No positive staining of CBX7 was detected in 10 normal gastric mucosal tissue samples (0/10, 0%). Compared with normal gastric mucosal tissues, gastric tumor tissues expressed significantly higher positive rate of CBX7 (p = 0.031).

### The correlation between the expression of CBX7 with clinicopathologic characteristics and prognosis

In paraffin-embedded archival gastric tumor samples, there was a significant positive correlation between CBX7 expression with clinical stage and lymph node metastasis (N classification), and a significant negative correlation between CBX7 expression and patients' age. The expression level of CBX7 was lower in patients with older age, and higher in patients with late clinical stage, or positive lymph node metastasis(Table 1), which suggested that overexpression of CBX7 correlated with a more aggressive phenotype in gastric cancer.

**Table 1:** The correlations between CBX7 expression and clinicopathologic variables, and p16 expression

Variables	CBX7 n (%)		
		
	(-)	(+)	P value*
**Gender**			
Male	34(68.0)	16(32.0)	
Female	16(64.0)	9(36.0)	0.729
**Age (years)**			
<60	15(50.0)	15(50.0)	
≥60	35(77.8)	10(22.2)	0.012
**Size(cm)**			
<4.5	26(65.0)	14(35.0)	
≥4.5	24(68.6)	11(31.4)	0.743
**Histology**			
Well differentiated	22(71.0)	9(29.0)	
Poorly differentiated	28(63.6)	16(36.4)	0.507
**T classification**			
T1/2	19(76)	6(24)	
T3/4	31(62.0)	19(38.0)	0.605
**LNM**			
Negative	31(77.5)	9(22.5)	
Positive	19(54.29)	16(45.71)	0.035
**Distant metastasis**			
Negative	48(82.76)	21(17.24)	
Positive	2(56.52)	4(43.48)	0.071
**Clinical stage**			
I/II	24(84.6)	5(15.4)	
III/IV	26(60.0)	20(40.0)	0.02
**p16**			
Negative	18(58.1)	13(41.9)	
Positive	32(72.7)	12(27.3)	0.188

Abbreviations: LNM, lymph node metastasis. *Data were analyzed by the χ2-test and p < 0.05 was considered to be significant.

**Figure 2:**
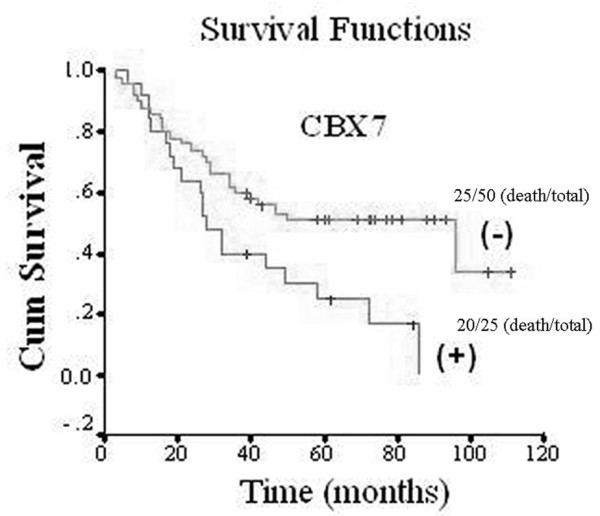
**CBX7 expression in gastric cancer tissues correlated with prognosis in univariate analysis**. Kaplan-Meier survival curves were plotted as cumulative survival vs months according to CBX7 expression (negative and positive).

All the patients were followed up to get the survival data. The median follow-up time was 52 months, and forty five patients had died at the last follow-up time. The 5-year overall survival rate in patients with positive CBX7 expression was significantly lower than those with negative CBX7 expression (25.0% vs. 35.0%, p < 0.001. Fig 2). The results suggest that overexpression of CBX7 correlates with poor prognosis in patients with gastric cancer. However, multivariate Cox proportional hazards model analyses, which included age, lymph node metastasis, distant metastasis, clinical stage, CBX7 protein expression and p16(INK4a) protein expression, showed that only lymph node metastasis was an independent prognostic indicator of overall survival, while CBX7 wasn't the independent prognostic indicator (Table 2).

**Table 2:** Multivariate analysis of prognostic factors by the Cox proportional hazards model in gastric carcinoma.

Variables	Hazard Ratio	95%CI	P value
Lymph node metastasis	4.201	1.120-15.762	0.033*
Clinical stage	1.869	0.818-4.268	0.138
CBX7	1.323	0.678-2.584	0.412
P16	1.265	0.696-2.299	0.440
Age	1.009	0.984-1.035	0.472
Distant metastasis	1.801	0.682-4.758	0.235

* p < 0.05 was considered to be significant

### Knockdown of CBX7 expression induces senescence and inhibits proliferation and migration of gastric cancer cells

**Figure 3:**
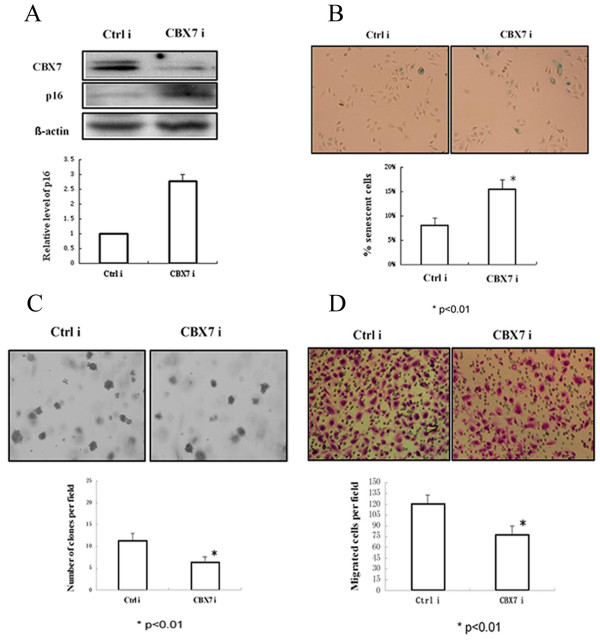
**Reduction of transformed phenotype by knockdown of CBX7 expression**. **A) **CBX7 knockdown in SGC-7901 cells resulted in the upregulation of p16 as determined by Western blot analysis. β-actin was used as a loading control. The level of p16 was quantified by densitometric analysis of signal present in each lane and normalizing it to β-actin signal of respective lane using ImageJ 1.37v software (NIH, Bethesda, USA). **B) **Knockdown of CBX7 expression in SGC-7901 cells resulted in increased cellular senescence (p < 0.01; upper panel, pictures of SA-β-gal stained cells; lower panel, senescent cells were counted and plotted). **C) **Decreased number of colonies in soft agar in CBX7 knockdown cells (p < 0.01; upper panel, pictures of colonies in soft agar; lower panel, the number of colony were counted and plotted). **D) **Transwell migration assays using the Corning chamber showed that fewer number of cells migrated in SGC-7901 cells with CBX7 knockdown compared with that in control (p < 0.01; upper panel, pictures of migrated cells; lower panel, the number of migrated cells were counted and plotted).

We determined the transformation potential of control and CBX7 knockdown SGC-7901 cells using anchorage-independent growth assay, and determined the senescence by SA-β-gal staining. Western blot analysis of CBX7 indicted that CBX7 siRNA efficiently knockdowned CBX7 expression (Fig 3A). Stable expression of CBX7 siRNA in SGC-7901 cells led to an increase of senescence and a decrease in colony formation in soft agar (Fig 3B, C). Compared to control, the rate of senescent cells was higher in CBX7 knockdown SGC-7901 cells (Fig 3B), and the soft agar colonies in CBX7 knockdown SGC-7901 cells were less in frequency and also smaller in size (Fig 3C).

As the expression of CBX7 in gastric cancer tissue samples correlated with lymph node metastasis, we hypothesized that CBX7 might also regulate cancer metastasis. In support of this hypothesis, we used an *in vitro *transwell chamber cell migration model to measure the effect of CBX7 on cell migration, which is one of the important steps in cancer metastasis. Results showed that the number of migrated cells decreased significantly in CBX7 knockdown SGC-7901 cells, compared to that in control cells (Fig 3D). Our results suggest that CBX7 regulates cell migration and that overexpression of CBX7 may contribute to cancer metastasis.

### p16(INK4a) is a target of CBX7 and may be one of the mechanisms

To determine the possible mechanisms of CBX7 in gastric carcinogenesis, we studied the relationship between CBX7 and p16(INK4a), which is a down-stream target of CBX7 during its controlling human normal cells lifespan. Firstly, we analyzed the expression of p16(INK4a) in gastric cancer cell lines and gastric cancer tissues. We found that the normal immortal human gastric mucosal epithelial cell line GES-1 expressed high level of p16(INK4a); while 3 of 8 gastric cancer cell lines expressed lower level of p16(INK4a), and 5 of 8 gastric cancer cell lines did not express detectable p16(INK4a). Cell lines with low or no p16(INK4a) overexpressing CBX7 suggested a negative correlation between the expression of CBX7 and p16(INK4a) (Fig 1A). However, we found the correlation between the expression of CBX7 and p16(INK4a) in gastric cancer tissue samples by IHC analyses was not significant (Table 1). Then, we examined the expression of p16(INK4a) in control and CBX7 knockdown SGC-7901 cells to determine the possible mechanism of decreased transformed phenotype in gastric cancer cell lines by knockdown of CBX7 expression. We found that knockdown of CBX7 resulted in increased p16(INK4a) expression (Fig 3A).

## Discussion

More and more studies revealed that different PcG proteins were involved in carcinogenesis and neoplastic progression. Bmi-1, as one of the best known PcG genes, plays an important role in regulating cellular proliferation, cellular senescence, tumorigenesis and functions as an oncogene [2-10]. Previous studies found that *INK4A/ARF *locus and AKT/PKB pathway are two important cancer relevant target of Bmi-1 in gastric and breast cancers [8,10]. It was found that CBX7 shares some similarities in functions and mechanisms with Bmi-1 including inhibiting cellular senescence and extending the lifespan of normal human cells via downregulating the expression of *INK4a/ARF *tumor suppressor locus [17,20,21]. Otherwise, CBX7 can initiate T-cell lymphomagenesis and cooperate with c-Myc to produce highly aggressive B-cell lymphomas in the generation of transgenic mice overexpressing CBX7 [11]. Moreover, it has also been shown that CBX7 expression facilitates the survival of the mouse embryonic fibroblasts [20]. These results suggest that CBX7 is also involved in carcinogenesis and acts as an oncogene like Bmi-1.

However, several recent publications propose CBX7 as a potential tumor suppressor. It was found that Loss of CBX7 expression correlated with a more aggressive phenotype in thyroid carcinoma, pancreatic adenocarcinoma and colorectal carcinoma [12-14]. The opposite role of CBX7 in different studies may be due to the different cancer types. Till now, studies concerning CBX7 are limited and the functions and mechanisms of CBX7 in caicinogenesis are still unclear. Its role in other cancer types including gastric cancer needs to be clarified.

Recently we reported that Bmi-1 was overexpressed in gastric cancer cell lines and gastric tumors and plays an important role in the carcinogenesis and progression of gastric cancer [10]. The function of CBX7 in the carcinogenesis and progression of gastric cancer needs to be studied. In the present study we provide *in vitro *and *in vivo *evidences to support the oncogenic role of CBX7 in gastric cancer development. Here we are the first time to show that CBX7 is overexpressed in gastric cancer cell lines and gastric cancer tissues; and stable knockdown of CBX7 expression in gastric cancer cells can induce cellular senescence, which constitutes a powerful barrier to oncogenesis [4], and inhibit proliferation in *in vitro *study. Importantly, we found that overexpression of CBX7 correlated with advanced clinical stage and positive lymph node metastasis. Our *in vitro *study also showed that knockdown of CBX7 expression inhibited the ability of migration in gastric cancer cells. This is the first time to find that CBX7 regulates cellular migration in *in vitro *model, and provide preliminary direct evidence for the possibility of CBX7 regulating the metastasis of cancer. All these results suggest that CBX7 not only play important roles in tumorigenesis, but may also be involved in the progression and metastasis of gastric cancer. Our previous study showed that Bmi-1 was an independent negative prognosis factor and patients with high Bmi-1 expression survived significantly shorter than those with low and no Bmi-1 expression [10]. In the present study, using the same patient samples, we also found that patients with positive CBX7 expression survived significantly shorter than those with negative CBX7 expression. However, multivariate Cox proportional hazards model analysis showed that lymph node metastasis, but not CBX7 is an independent prognosis factor. Collectively, our data suggest CBX7 shares similarities in functions with Bmi-1 in gastric cancer, but we didn't confirm CBX7 is an independent prognosis factor as Bmi-1, which may be due to the limited samples in the present study, or the function of CBX7 may partially depend on Bmi-1, or its role is not as important as Bmi-1 in gastric cancer.

It is interesting to note that the expression of CBX7 negatively correlated with age in this study. The positive expression rate of CBX7 in old patients was significantly lower than that in young patients. As CBX7 is capable of regulating cellular proliferation and senescence [20], and CBX7 expression is downregulated during replicative senescence, the results suggest that cancer cells in aged person might have lower proliferative ability, or more cells in aged person are in the senescent state.

It's already known that CBX7 regulates cellular senescence and proliferation via *Ink4a/Arf *locus, which encodes the cyclin-dependent kinase inhibitor p16(INK4a) and tumor suppressor p19(Arf) [20]. However, what's the down-stream target and mechanism of CBX7 during gastric carcinogenesis is still unclear. In the present study we found that knockdown of CBX7 resulted in increased p16(INK4a) expression and was accompanied by decreased transformed phenotype and migration ability, which suggested regulation of p16(INK4a) might be one of the important mechanisms of CBX7 in gastric cancer. However, in our *in vivo *study we did not find correlation between CBX7 and p16(INK4a) expression in gastric tumor tissues. The discrepancy could be due to the limited number of samples in our study, or other co-exist genes regulating p16(INK4a) and promoter methylation induced loss of p16(INK4a) expression might interfere and influence the results of correlation analysis. So the mechanisms of CBX7 in gastric cancer still need to be further studied.

## Conclusions

CBX7 plays a role in the carcinogenesis and progression of gastric cancer and acts as an oncogene, and it may regulate tumorigenesis, cell migration and cancer metastasis partially via p16(INK4a) regulatory pathway.

## Abbreviations

PcG: Polycomb group; RNAi: RNA interference; CBX7 i: CBX7 siRNA; Ctrl i: control siRNA; SA-β -gal: Senescence-associated beta-galactosidase; IHC: Immunohistochemical

## Competing interests

The authors declare that they have no competing interests.

## Authors' contributions

XWZ, LZ contributed equally to the experiments, data analysis and interpretation of data; WJG made contributions to the study design; WQ, XHY, XL, LZZ contributed to the experiments; JL made contributions to the study design; XWZ drafted the article and WJG revised it. All the authors have read and approved the final manuscript.
